# Inflammatory Response to Traumatic Injury: Clinical and Animal Researches in Inflammation

**DOI:** 10.1155/2015/729637

**Published:** 2015-07-28

**Authors:** Huang-Ping Yu, Irshad H. Chaudry, Mashkoor A. Choudhry, Chung-Hsi Hsing, Fu-Chao Liu, Zhengyuan Xia

**Affiliations:** ^1^Department of Anesthesiology, Chang Gung Memorial Hospital, Taoyuan 333, Taiwan; ^2^College of Medicine, Chang Gung University, Taoyuan 333, Taiwan; ^3^Department of Surgery and Center for Surgical Research, University of Alabama at Birmingham, Birmingham, AL 35294, USA; ^4^Department of Surgery, Loyola University Chicago Health Sciences Campus, Maywood, IL 60153, USA; ^5^Department of Anesthesiology, Chi Mei Medical Center, Tainan 710, Taiwan; ^6^Department of Anesthesiology, University of Hong Kong, Pokfulam, Hong Kong

Inflammatory response is usually associated with various traumatic insults. In addition, traumatic injury produces excessive proinflammatory mediators and subsequent activating or recruiting immune cells into the target organs and results in systemic inflammatory response. However, the complex pathways and mechanisms of inflammatory responses on traumatic injury have not been clearly elucidated. The aim of this special issue is to describe the role of inflammation in traumatic injury, providing an up-to-date viewpoint for therapeutic approaches.

Patients after tangential excision surgery usually suffered from tremendous pain, which leads to anxiety, emergency, agitation, and inflammatory reaction. W. Geng et al. showed that preoperative administration of flurbiprofen axetil decreased postoperative tramadol consumption and the visual analog scale after surgery. In addition, flurbiprofen axetil attenuated emergency agitation score and Ramsay score after extubation and reduced the systemic levels of proinflammatory cytokines tumor necrosis factor-*α* and interleukin-6 after the operation. The adverse events induced by intubation and extubation may cause intracranial hemorrhage and increase of intracranial pressure, especially in posterior fossa surgery patients. C. Tang et al. suggested that I-gel combined with tracheal intubation could reduce the stress response of posterior fossa surgery patients. Plasma *β*-endorphin, cortisol, interleukin-6, tumor necrosis factor-*α*, malondialdehyde concentrations, and blood glucose were decreased in I-gel facilitated endotracheal tube intubation group. Utilization of I-gel combined with endotracheal tube in posterior fossa surgery may lower inflammatory and oxidative response.

Y. Chen et al. reported that sepsis-induced acute intestinal injury was attenuated by dexmedetomidine treatment. The protective effects of dexmedetomidine on sepsis-induced injury may be associated with the inhibition of inflammation via modulating toll-like receptor 4 pathway. Inhalation anesthetics isoflurane may inhibit hypoxia pulmonary vasoconstriction. In addition, dexmedetomidine can reduce the dose of isoflurane inhalation and potentiate hypoxia pulmonary vasoconstriction. R. Xia et al. reported that dexmedetomidine could augment hypoxia pulmonary vasoconstriction and improve oxygenation during one-lung ventilation through inhibiting oxidative stress and increasing nitric oxide release. Histone deacetylases modulate cytokine synthesis and release. Trichostatin A, a histone deacetylase inhibitor, is documented to be anti-inflammatory and neuroprotective. C.-H. Hsing et al. indicated that trichostatin A reduced lipopolysaccharide-induced neuroinflammation and cognitive dysfunction. J.-A. Lin et al. first identified the effects of bone components on toll-like receptor 4 surface expression. Toll-like receptor 4 surface expression in human monocytes was used as the main target to assess immune dysfunction following bone component exposure. They found that bone component exposure downregulated toll-like receptor 4 surface expression. Perinatal insults and subsequent neuroinflammation is the major mechanism of neonatal brain injury. C.-Y. Chen et al. reported that hypoxic preconditioning conferred strong neuroprotection, likely through suppression of glial activation and subsequent inflammatory responses after hypoxia-ischemia insults in neonatal rats.

The elderly hip fracture is one of the most common injuries and represents a high incidence of complications and mortality. L. Gan et al. examined the influence of immediate surgery on systemic inflammatory response and lung injury induced by elderly hip fracture. They found that the elderly hip fracture could result in systemic inflammatory response and lung injury. In addition, the proximal femoral intramedullary pinning surgery following hip fracture aggravated the above pathological states by increasing the mitochondrial DNA release. Cardiopulmonary bypass induces neutrophil activation and release of matrix metalloproteinases-9, contributing to postoperative pulmonary infiltration and dysfunction. T.-C. Lin et al. reported that elective cardiac surgery with cardiopulmonary bypass induced short-term elevation of plasma metalloproteinases-9 concentrations within 24 hours. However, increase of neutrophils and reduced oxygenation are not correlated with cardiopulmonary bypass time and postoperative pulmonary dysfunction. Y. Tian et al. suggested that combination of 3-day captopril treatment and isoflurane preconditioning additively attenuated myocardial ischemia reperfusion by reducing oxidative stress and inflammation. Acute kidney injury is a frequent and severe complication during sepsis. Though fluid resuscitation is the main therapy, heart failure is usually lethal for those patients receiving large volumes of fluids. Y. Wang et al. reported that single small-volume resuscitation with hydroxyethyl starch and hypertonic saline hydroxyethyl starch could attenuate endotoxemia-induced kidney injury.

Resveratrol, a natural polyphenolic compound of grape and red wine, owns potential anti-inflammatory effects, which results in the reduction of cytokines overproduction, the inhibition of neutrophil activity, and the alteration of adhesion molecules expression. Resveratrol has been shown to reduce organ damage following traumatic and shock-like states. Such protective phenomenon is reported to be implicated in a variety of intracellular signaling pathways including the activation of estrogen receptor, the regulation of the sirtuin 1/nuclear factor-kappa B and mitogen-activated protein kinases/hemeoxygenase-1 pathway, and the mediation of proinflammatory cytokines, reactive oxygen species formation and reaction. F.-C. Liu et al. reviewed the organ-protective and anti-inflammatory effects of resveratrol in trauma-hemorrhagic injury. S. Hamaguchi et al. reported elevated circulating free DNA levels during early-phase sepsis might represent a candidate biomarker for the severity of sepsis. In addition, regulatory T cells suppress excessive immune responses and are potential therapeutic targets in autoimmune disease and organ transplantation rejection. C. Jun et al. found that recruitment of regulatory T cells into the kidney was related to the recovery of renal function after ischemia–reperfusion injury.

Together, the reviews, research articles, and clinical studies that are featured in this special issue enhance our knowledge base of inflammation in traumatic injury.

*Huang-Ping Yu* *Huang-Ping Yu* *Fu-Chao Liu* *Fu-Chao Liu* *Chung-Hsi Hsing* *Chung-Hsi Hsing* *Zhengyuan Xia* *Zhengyuan Xia* *Mashkoor A. Choudhry* *Mashkoor A. Choudhry* *Irshad H. Chaudry* *Irshad H. Chaudry*

